# An Overview of the Current Known and Unknown Roles of Vitamin D_3_ in the Female Reproductive System: Lessons from Farm Animals, Birds, and Fish

**DOI:** 10.3390/ijms232214137

**Published:** 2022-11-16

**Authors:** Malgorzata Grzesiak, Marcelina Tchurzyk, Magdalena Socha, Andrzej Sechman, Anna Hrabia

**Affiliations:** 1 Department of Endocrinology, Institute of Zoology and Biomedical Research, Jagiellonian University in Krakow, Gronostajowa 9, 30-387 Krakow, Poland; 2 Department of Animal Physiology and Endocrinology, University of Agriculture in Krakow, al. Mickiewicza 21, 31-120 Krakow, Poland

**Keywords:** vitamin D_3_, ovary, uterus, farm animals, birds, fish

## Abstract

Recent studies have clearly shown that vitamin D_3_ is a crucial regulator of the female reproductive process in humans and animals. Knowledge of the expression of vitamin D_3_ receptors and related molecules in the female reproductive organs such as ovaries, uterus, oviduct, or placenta under physiological and pathological conditions highlights its contribution to the proper function of the reproductive system in females. Furthermore, vitamin D_3_ deficiency leads to serious reproductive disturbances and pathologies including ovarian cysts. Although the influence of vitamin D_3_ on the reproductive processes of humans and rodents has been extensively described, the association between vitamin D_3_ and female reproductive function in farm animals, birds, and fish has rarely been summarized. In this review, we provide an overview of the role of vitamin D_3_ in the reproductive system of those animals, with special attention paid to the expression of vitamin D_3_ receptors and its metabolic molecules. This updated information could be essential for better understanding animal physiology and overcoming the incidence of infertility, which is crucial for optimizing reproductive outcomes in female livestock.

## 1. Introduction

In mammals, birds, and fish, the proper female reproductive functions are dependent on the actions of pituitary gonadotropins, steroid hormones synthesized in the reproductive organs, and locally produced factors acting as paracrine/autocrine signals within the female reproductive tract [1,2,3,4,5,6,7]. Among the variety of newly described factors regulating reproduction in females, vitamin D_3_ has recently been extensively studied, especially in relation to humans (for review see [8,9,10]). Although vitamin D_3_ is known for its role in calcium-phosphorus homeostasis, there is a growing concern that global vitamin D_3_ deficiency/insufficiency contributes to serious reproductive disturbances and pathologies, including ovarian cysts, premature ovarian failure, uterine fibroids, and cancer in women [11,12,13]. It is noteworthy that, environmental (season, latitude, and nutrition), genetic, and hormonal factors affect vitamin D_3_ concentration not only in humans but also in animals [14,15,16]. For example, modern swine production that limits exposure to natural sunlight leads to an insufficient vitamin D_3_ concentration despite dietary supplementation [17]. Furthermore, indoor breeding pigs have a lower birth rate and litter size than grazing ones, which correlates with a low plasma vitamin D_3_ level [18]. Research performed on wild Soya sheep provides evidence that vitamin D_3_ status improves female reproductive performance and that natural selection acts on vitamin D_3_ metabolism [14]. Additionally, hens fed a vitamin D_3_-deficient diet lay eggs with thinner shells as well as numerous thin-shelled and soft-shelled eggs, but subsequent vitamin D_3_ administration restores normal egg production [19]. In humans and rodents, the effect of vitamin D_3_ on female reproductive processes has been described in several reviews [11,12,20,21,22,23,24,25,26]. However, its role in the reproduction of female livestock, including farm animals, birds, and fish, has rarely been summarized. Therefore, this review was prepared to present the known and unknown roles of vitamin D_3_ within the reproductive system of these animals, paying special attention to the expression of vitamin D_3_ receptors and metabolic molecules (Table 1).

## 2. Vitamin D_3_ Metabolism—Comparative Aspects

**Figure 1:**
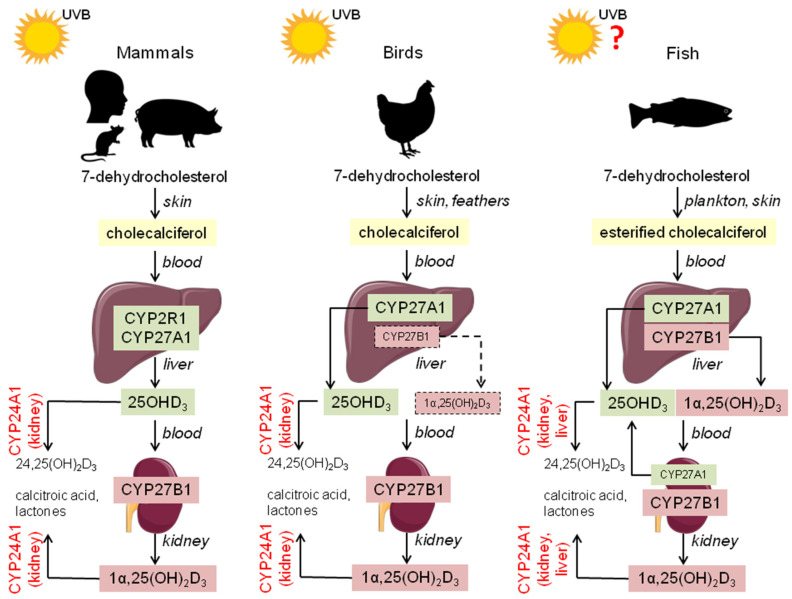
General pathways of vitamin D_3_ metabolism in mammals, birds, and fish. Abbreviations: CYP2R1 and CYP27A1= 25-hydroxylases; CYP27B1 = 1α-hydroxylase; CYP24A1 = 24-hydroxylase; UVB = ultraviolet B irradiation; ? = knowledge about synthesis is inconsistent.

The vitamin D molecule has been synthesized by organisms since early in the evolutionary history of animals. This capability might have appeared about 1.2 billion years ago [40]. In early marine species, such as zooplankton and phytoplankton, sunlight mediated the production of vitamin D, which probably protected against DNA damage from ultraviolet B (UVB) exposure [41,42]. Vitamin D refers to a group of fat-soluble secosteroids, and its two major forms are vitamin D_2_ (ergocalciferol) and vitamin D_3_ (cholecalciferol). In humans and animals, vitamin D_3_ is sourced through diet or an endogenous production in the skin [16]. In some avian species with uropygial glands, 7-dehydrocholesterol (7-DHC) is present in the oil that is spread over the feathers and is further metabolized [16]. In fish, vitamin D_3_ is only or mostly sourced from the aquatic food chain (zooplankton and phytoplankton) [43,44], but the possibility of cutaneous synthesis from 7-DHC esterified with long-chain fatty acids was also indicated in rainbow trout (*Oncorhynchus mykiss*) [45]. In keratinocytes, 7-DHC is converted to pre-vitamin D_3_ by breaking a B ring upon UVB irradiation. Next, this thermo-sensitive molecule can be easily isomerized to vitamin D_3_ (cholecalciferol) by exposure to body heat [46]. Subsequently, vitamin D_3_ is transported through the bloodstream to the liver and then to the kidneys, where it is hydroxylated by a subfamily of cytochrome P450 enzymes (CYPs) to other vitamin D_3_ metabolites [47]. Transport of vitamin D_3_ in the blood system occurs mostly in the presence of vitamin D binding protein (VDBP), and a much smaller amount can be bound to albumin or circulates as a free molecule [16]. The first step of vitamin D_3_ hydroxylation takes place in the liver under 25-hydroxylase’s action; CYP2R1 was found in humans and CYP27A1 in non-human species, which convert cholecalciferol into 25OHD_3_ (25-hydroxyvitamin D_3_; calcidiol). Next, the hydroxylation of 25OHD_3_ in the kidneys leads to the production of biologically active 1α,25(OH)_2_D_3_ (1α,25-dihydroxyvitamin D_3_; calcitriol). This process is induced by 1α-hydroxylase (CYP27B1) [40], which was also found in several non-renal tissues in mammals, i.e., intestine, lung, skin, thyroid, osteoblasts and chondrocytes, immune system cells, and reproductive organs [48]. There are some differences in vitamin D_3_ metabolism in fish. In most species, both hydroxylations of vitamin D_3_ can take place in the liver without the blood transport of 25OHD_3_ to the kidney, as in mammals [44]. On the other hand, in sea bream (*Sparus auratus*), this step occurs mostly in the kidneys [49]. Generally, the plasma level of 25OHD_3_ in fish is low as compared to mammals, while the concentration of 1α,25(OH)_2_D_3_ is much higher in freshwater common carp (*Cyprinus carpio*) and marine fish such as Atlantic salmon (*Salmo salar*) [50,51]. Importantly, there is evidence indicating novel pathways of vitamin D_3_ metabolism initiated by cytochrome P450scc (CYP11A1) in the placenta, adrenal glands, and epidermal keratinocytes that can be modified depending on CYP27B1 activity [52,53]. The circulating vitamin D_3_ metabolites 25OHD_3_ and 1α,25(OH)_2_D_3_ can be inactivated by 24-hydroxylase (CYP24A1) to 24,25(OH)_2_D_3_ and calcitroic acid, respectively [54]. Depending on the species, CYP24A1 may also exhibit 23-hydroxylase activity and catalyze the production of biologically active lactones [55]. The comparative aspects of vitamin D_3_ metabolism in mammals, birds, and fish are shown in Figure 1.

## 3. Genomic and Non-Genomic Action of Vitamin D_3_

Vitamin D_3_ is a secosteroid that can penetrate cellular membranes and bind to their nuclear receptors (VDR), acting as a transcription factor and triggering genomic action of 1α,25(OH)_2_D_3_ in cells [54,56]. The first known species to express VDR with a high affinity to 1α,25(OH)_2_D_3_ was a sea lamprey (*Petromyzon marinus*). In fact, since VDR appeared in this basal vertebrate species about 550 million years ago, other vertebrates from bony fish to mammals have also expressed VDR with the ability to interact with 1α,25(OH)_2_D_3_ [40]. Despite the free hormone hypothesis that steroids enter target cells by passive diffusion, transmembrane proteins such as cubilin [57] and megalin/LRP2 (LDL receptor-related protein 2) [58] were suggested as participating in the endocytosis of 25OHD_3_ bound with DBP, affecting its cellular uptake.

VDR consists of three domains: a NH_2_-terminal binding domain (DBD), a COOH-terminal binding domain (LBD), and a hinge region that serves as a link between them [54]. The highly conserved DBD is responsible for the recognition of specific DNA sequences named vitamin D response elements (VDREs), which are mainly located in the promoter region of genes and consist of hexamers separated by any three nucleotides [59]. The LBD is necessary to bind the ligand and to interact mostly with the retinoid X receptor (RXR), forming a VDR-RXR heterodimer [46]. It was demonstrated that LBD possesses two overlapping binding sites: the VDR-genomic pocket and the VDR-alternative pocket, which mediate genomic and non-genomic responses, respectively [23,60]. The VDR-RXR heterodimeric complex also recruits co-regulatory proteins that induce epigenetic changes such as histone acetylation, deacetylation, or methylation, and results in either activation or suppression of gene transcription [23]. Besides its presence in the cytoplasm/nucleus, VDR was also found in the plasma membranes, including caveolae [61], mitochondria membranes [62], and lipid droplets [63]. From the above mentioned cellular VDR localization and the presence of the VDR-alternative pocket in LBD taken together, the involvement of VDR in rapid responses was postulated as well [64].

Apart from genomic action, 1α,25(OH)_2_D_3_ can exert its biological effect by activating a rapid non-genomic pathway [56]. However, this response is much less understood. The most known protein probably responsible for a rapid cellular response to vitamin D_3_ is PDIA3 (protein disulfide isomerase family A member 3), previously named MAARS (1α,25(OH)_2_D_3_; membrane-associated rapid response to steroid) or ERp57 [64]. The induction of the non-genomic pathway requires the interaction of PDIA3 with caveolin 1 (CAV1), a protein present in the membrane caveolae [47]. This can cause the activation of phospholipase A2 (PLA2), phospholipase C (PLC), protein kinase C (PKC), and Wnt family member 5A (WNT5A) pathways. Additionally, mitogen-activated protein kinases (MAPK) and phosphatidylinositol-3 kinase (PI3K) signaling pathways can be induced by PDIA3 [56,64]. 1α,25(OH)_2_D_3_-mediated non-genomic actions could affect some genomic actions as well, but these pathways still require further study [47,56].

As noted above, VDR has been also introduced as a membrane receptor for vitamin D_3_ that can cause non-genomic actions. Membrane-associated VDR (VDRm) might activate several pathways through its interaction with CAV1, including the SRC (SRC proto-oncogene, non-receptor tyrosine kinase) pathway. The VDR-triggered downstream effect is associated with the regulation of the transcriptional activity of WNT, NOTCH, and the sonic hedgehog signaling molecule (SHH) signaling pathways [56].

## 4. Vitamin D_3_ Action in the Female Reproductive System of Farm Animals

The mammalian female reproductive tract consists of the ovaries and the duct system, including oviducts, uterus, cervix, vagina, and external genitalia. The paired ovaries produce the oocytes, which are released during ovulation. They are transported to the oviducts, where fertilization may occur, and then to the uterus, where the blastocyst can implant into the thickened uterine endometrium and develop during pregnancy [65]. Recent studies have shown that mammalian reproductive organs, such as ovaries, uterus, oviducts, or placenta, are important extra-renal sites of vitamin D_3_ metabolism and action (for review see [24,26]). Results from knockout mice revealed that deficiencies of the *Vdr* and *Cyp27b1* genes led to impaired folliculogenesis, lack of corpora lutea, and uterine hypoplasia [66,67,68], providing evidence for the indispensable role of vitamin D_3_ and its receptor in the development and function of the female reproductive system. Considering that farm animals are agriculturally important species, a summary of updated knowledge on vitamin D_3_ action and the expression of its related molecules within the female reproductive organs could be important for optimizing reproductive outcomes.

### 4.1. Ovary

The most important functional unit of the ovary is the ovarian follicle, which supports oocyte maturation and synthesizes steroid hormones as well as local factors that are critical for successful reproduction [69]. The VDR mRNA transcript and protein abundance have been described in porcine [28,29] and caprine [27,70] ovarian follicles, indicating the genomic action of vitamin D_3_ within the ovary of those domestic animals. VDR immunolocalization was shown in granulosa and theca cells of healthy antral follicles [27,29,70] and atretic follicles [70], and in oocytes of primordial and primary ones [60]. In addition, a recent study by Grzesiak et al. [29] also suggested the plausible non-genomic response of porcine antral follicles to vitamin D_3_ through the membranous protein PDIA3, which was immunolocalized in granulosa and theca cells and was detected at the transcript and protein levels. 

In pigs, the ovarian granulosa and theca interna cells were found to have the capacity to locally metabolize vitamin D_3_ due to the presence of CYP27B1 and CYP24A1 enzymes [29]. Besides their specific localization in follicular compartments, their mRNA transcript and protein abundance was detected in antral follicles at all developmental stages. Furthermore, the presence of 1α,25(OH)_2_D_3_ in the follicular fluid of antral follicles indicates possible local ovarian vitamin D_3_ production [29]. Both the expression of vitamin D_3_ receptors and metabolic enzymes depend on follicle size; VDR and PDIA3 mRNA transcript and protein abundance decreased with antral follicle growth, while the CYP27B1 mRNA transcript and protein, and *CYP24A1* mRNA transcript abundances were greatest in medium antral follicles [29]. On the contrary, in goats VDR mRNA transcript and protein abundance correlated with follicle size [70]. This indicates the possible species-specific regulation of ovarian follicle development by vitamin D_3_. It is noteworthy that, impaired vitamin D_3_ metabolism was marked by decreased abundances of CYP27B1 and CYP24A1 proteins, and a diminished 25OHD_3_ concentration in the fluid was found in the follicular and follicular lutein cysts of sows in comparison to preovulatory follicles [71]. Overall, vitamin D_3_ and its related molecule system seem to be important in the proper course of folliculogenesis in farm animals. 

Follicle growth and development are tightly coordinated through continuous cell proliferation and apoptosis [72]. In goats, vitamin D_3_ (0, 1, 10 and 100 nM) was found to induce granulosa cell proliferation in a dose-dependent manner by regulation of cell cycle- and cellular oxidative stress-related genes [27,70]. In detail, vitamin D_3_ at a dose of 10 nM induced cell cycle arrest from the G0/G1 to S phases through the upregulation of *CDK4* and *CyclinD1* and the downregulation of *P21* genes. Furthermore, Yao et al. [27] showed significantly decreased production of reactive oxygen species, which can inhibit cell proliferation, in response to vitamin D_3_ (10 nM) and increased the expression of free radical scavenging enzymes such as catalase and sodium dismutase 1 within *in vitro* caprine granulosa cell cultures. Regarding the involvement of vitamin D_3_ in follicular apoptosis, *VDR* silencing in goat granulosa cells resulted in the increased abundance of the pro-apoptotic BAX protein and the decreased abundance of the anti-apoptotic Bcl-2 protein [70]. As expected, VDR overexpression exerted the opposite effect manifested by increasing the anti-apoptotic protein level [70]. Taken together, after binding to VDR, vitamin D_3_ could modulate ovarian folliculogenesis via the regulation of cell cycle- and apoptosis-related genes.

The ovary is an important endocrine gland, producing sex steroid hormones in response to pituitary gonadotropins [69]. Thus far, research has reported the impact of vitamin D_3_ on the steroidogenic pathway in the follicular cells of farm animals [27,29,73,74,75]. Vitamin D_3_ (10 nM) increased the secretion of progesterone by caprine granulosa cells *in vitro* through the upregulation of *StAR* and *3β-HSD* genes [27] as well as by small and medium antral porcine follicles harvested from sexually mature gilts (10 and 50 ng/mL) [29]. On the contrary, other studies conducted on porcine granulosa cells described elevated FSH- and insulin-induced progesterone secretion, but there was no effect on its basal release [73] or reduced progesterone secretion by granulosa cells isolated from small antral follicles of immature pigs upon vitamin D_3_ treatment (100 ng/mL and 100 nM, respectively) [74]. There is more consistent evidence for the influence of vitamin D_3_ on follicular estradiol production; the stimulatory effect was observed in caprine granulosa cells (10 nM) [27] and in porcine granulosa cells (100 ng/mL) [73,74], and in small (100 ng/mL) and medium (1–100 ng/mL) antral follicles of mature gilts [29]. These results are not surprising due to the presence of VDRE in the promoter region of the gene encoding CYP19A1, which converts androgens to estrogens [76]. Besides the generally accepted genomic effect of vitamin D_3_ on estradiol secretion through the upregulation of the *CYP19A1* gene, its non-genomic action was also speculated. In goats, enhanced estradiol synthesis was not accompanied by increased *CYP19A1* mRNA transcript abundance in granulosa cells, but the intracellular cAMP level was elevated [27]. Little is known about the role of vitamin D_3_ in the regulation of androgen production in the ovary. The only study on pigs showed unchanged testosterone release by antral follicles *in vitro* upon vitamin D_3_ treatment (1–100 ng/mL) [29]. On the other hand, testosterone was found to affect the transcriptional activity of VDR in porcine granulosa cells by inhibiting the formation of VDR-RXR complexes [28].

To sum up, the ovarian follicle was confirmed to be a target tissue for vitamin D_3_ action (genomic and/or non-genomic) and an extra-renal site of its metabolism. Furthermore, vitamin D_3_ appears to be a crucial intraovarian regulator of folliculogenesis and steroidogenesis in farm animals such as pigs and goats (Table 2), and may consequently influence reproductive efficiency.

### 4.2. Uterus

The mammalian uterus consists of two functional compartments, the endometrium and the myometrium. The endometrium comprises luminal and glandular epithelial cells as well as stromal cells and undergoes dynamic morphological and physiological changes during each estrous cycle and pregnancy [77]. As the ovarian follicle, the uterus is also a target tissue for potential vitamin D_3_ action. However, so far only a genomic response through VDR has been described. In detail, VDR was found in the cyclic uterus of buffalo cows [32] and pigs [33], and in the gravid uterus of pigs [34] and sheep [35]. Both the endometrium and the myometrium revealed VDR mRNA transcripts and protein abundance, but more specifically, VDR was immunolocalized in the luminal and glandular epithelium and in stromal and myometrial cells. It is noteworthy that, VDR level (endometrial in buffalo cow, and both endometrial and myometrial in pigs) varied depending on the day of the estrous cycle; the greatest abundance was observed during the mid-luteal phase, suggesting a regulatory role of progesterone, for which a high level was detected at that period [32,33]. Additionally, gestational days influenced endometrial *VDR* mRNA transcript abundance in pigs [34] and sheep [35], indicating the potentially important role of vitamin D_3_ in the establishment of pregnancy in livestock species. 

The uterus of farm animals is another reproductive tissue of vitamin D_3_ metabolism beyond the kidneys. The expression of related metabolic enzymes has been documented in cyclic and gravid uteri of pigs and sheep [34,35,39]. During the estrous cycle, the synthesizing enzyme CYP27B1 transcript and protein were detected in the endometrium and myometrium, while the inactivating enzyme CYP24A1 transcript and protein were found exclusively in the endometrium [39]. Furthermore, immunofluorescent localization of CYP27B1 was observed in luminal and glandular epithelial cells, stroma cells, and myocytes, whereas CYP24A1 was only found within the endometrial compartment [39]. In general, the highest CYP27B1 mRNA transcript and protein abundance and the *CYP24A1* mRNA transcript were noted in the follicular phase that corresponded with elevated 1α,25(OH)_2_D_3_ concentration in uterine flushings. These results suggest the plausible contribution of both the endometrium and the myometrium in the creation of intrauterine vitamin D_3_ milieu in pigs, for which an exact role has not been fully described in the estrous cycle. The only study by Grzesiak et al. [33] reported the positive effect of 1α,25(OH)_2_D_3_ at doses 10 and 50 ng/mL on estradiol secretion by myometrial explants of pigs, implicating its regulatory role in uterine steroidogenesis. Regarding pregnancy, porcine [34] and ovine [35] endometria were examined, and the *CYP2R1*, *CYP27B1*, and *CYP24A1* genes were assessed with some species-specific fluctuations at the stage of gestation. *CYP2R1* and *CYP27B1* mRNA transcript abundances were stable across gestation in sheep, whereas in pigs, *CYP2R1* gene expression was greater in late pregnancy and *CYP27B1* during mid-to-late gestation. In both examined species, *CYP24A1* gene expression was greatest during early pregnancy [34,35]. Since an increased calcitriol level in porcine endometrial tissue was observed on days 12 and 15 of gestation [70], the extensive 1α,25(OH)_2_D_3_ inactivation by CYP24A1 at that time could be required. Given that 1α,25(OH)_2_D_3_ was shown to upregulate *CYP24A1* mRNA transcript abundance in porcine endometrial explants [34,39], the local negative feedback mechanism to regulate endometrial calcitriol concentration within early pregnancy in farm animals was postulated. Since ewes’ pre-mating vitamin D_3_ status was associated with lamb birth weight, vitamin D_3_ was indicated as influencing fetal survival in the uterus [15].

Vitamin D_3_-modulated transport of calcium and phosphate during pregnancy is crucial for appropriate fetal growth, mainly by the effect on skeletal mineralization [78]. Indeed, the expression and localization of molecules involved in calcium binding and transport were detected in the maternal-conceptus interface of pigs and sheep [34,35]. Together with the elevated calcium concentration in uterine flushing during the peri-implantation period, these observations suggest a crucial role of calcium in conceptus growth and implantation in those farm animals [34,79]. The maternal-fetal interface also expresses vitamin D_3_-related molecules that seems to maintain intrauterine mineral homeostasis, but otherwise, vitamin D_3_ was shown to regulate other processes important for successful implantation [34,35]. In porcine endometrial explants, calcitriol affected the expression of implantation-related genes such as *FGF7*, *LPAR3*, *STC1*, and *SPP1* that are involved in the proliferation and differentiation of conceptus trophectoderm and cell-to-cell adhesion between conceptus and endometrium [34]. These genes are upregulated by estradiol secreted through the conceptus, thus its cooperation with vitamin D_3_ is plausible during the critical period of pregnancy recognition in pigs [34]. Along this line, interferon tau, which is the maternal pregnancy recognition signal in sheep, was reported to play a regulatory role together with progesterone in the metabolic inactivation of 1α,25(OH)_2_D_3_ by conversion to 1α,24,25(OH)_3_D_3_ in the ovine uterus [38]. Overall, these recent studies suggest an important role of vitamin D_3_ during the peri-implantation period in livestock species.

## 5. Vitamin D_3_ Action in the Female Reproductive System of Birds

The reproductive system of birds differs from that of mammals. In the majority of hens, only the left ovary and oviduct exist. In the ovary of mature hens, there are follicles at different stages of development. The most numerous are slow-growing prehierarchical follicles: primordial and primary (<1 mm in diameter in domestic chickens), white (>1–4 mm), and yellowish (>4–8 mm). The other group comprises more rapidly growing, yellow follicles (>8–40 mm), arranged into the preovulatory hierarchy. The largest follicle in a hierarchy (F1) is the most mature and will ovulate first [2,80]. The structure remaining after oocyte release is a postovulatory follicle, which undergoes tremendous regression within 5–6 days [81]. The released ovum is taken up by the oviduct where the constituents of the laid egg, including the egg white, eggshell membranes, and eggshell, are produced. The avian oviduct consists of five morphologically and functionally different parts: the infundibulum (engulfs the ovulated oocyte), magnum (synthesizes and secretes the majority of the egg albumen), isthmus (forms shell membranes), shell gland (deposits the calcified eggshell), and vagina (helps in egg expulsion) [7,82]. In contrast to mammals, much less is known about the participation of vitamin D_3_ in the local regulation of reproductive processes in hens; however, growing evidence demonstrates that vitamin D_3_ is involved in the avian reproductive system’s functioning as well. It is especially crucial for maintaining egg production and eggshell quality in hens. Vitamin D_3_ metabolism and regulation show large similarities in mammals and birds, but the magnitude of the fluctuation in hens is distinctly larger [83].

### 5.1. Ovary

The specific binding of vitamin D_3_ to VDR in the hen ovary has been demonstrated first by DNA cellulose chromatography, sucrose density gradient analysis, and saturation analysis, suggesting the potential direct action of vitamin D_3_ in this organ [84]. The presence of VDR transcripts was subsequently revealed in the granulosa layer of chicken ovarian follicles and their abundance increased with follicle development. Significantly higher mRNA abundance occurred in the granulosa layer cells of yellow follicles, 9–16 mm and F1, compared with granulosa cells of white follicles (3–5 mm). Immunohistochemically, VDR protein was localized primarily to nuclei of granulosa cells of the hen follicle [30]. The expression of RXR mRNA in all compartments of the chicken ovary, i.e., ovarian stroma, white, yellowish, yellow, and postovulatory follicles, has also been demonstrated [85]. These data may indicate the cooperation of VDR and RXR in signal transduction initiated by vitamin D_3_ in the hen ovary. 

Subsequent studies have shown that the role of vitamin D_3_ in the hen ovary may be associated with the regulation of steroidogenesis, cell proliferation, and expression of some genes related to follicle development. Namely, hens maintained on a vitamin D_3_-deficient diet with calcium supplementation ceased egg laying and had decreased ovarian weight and plasma estradiol and progesterone concentrations compared with those on a control diet [86]. In *in vitro* conditions, vitamin D_3_ increased the proliferation of granulosa cells collected from prehierarchical follicles, both white (3–5 mm) and yellowish (6–8 mm). Moreover, vitamin D_3_ at doses 10 and 100 nM decreased *AMH* mRNA transcript and at dose 100 nM increased *FSHR* mRNA transcript abundances [30] and stimulated *Kit ligand* mRNA transcript abundance [87] in granulosa cells of slow-growing follicles of the hen. From a group of 6–8 mm follicles, one is recruited daily into the preovulatory hierarchy, so vitamin D_3_ by alterations in the above gene expression around the time of follicle selection may be involved in this process’s regulation (Table 2). Thus far, there is no information regarding the influence of vitamin D_3_ on processes occurring in yellow preovulatory follicles; however, 9-cis retinoid acid has been demonstrated as a potent regulator of estradiol and progesterone synthesis and/or secretion from chicken ovarian follicles. It inhibits estradiol secretion by the theca cells and stimulates progesterone release by the granulosa cells [85]. Therefore, it cannot be excluded that retinoid acid seems to be part of the VDR-RXR signaling system in the avian ovary.

### 5.2. Oviduct

Another investigation showed that the chicken oviduct, like the ovary, is a target organ for vitamin D_3_. Within the oviductal parts, the role of vitamin D_3_ has been mostly elucidated in the shell gland (uterus), where it seems to play an especially important role in eggshell formation [83]. The VDR [36,37] and VDBP [88] are present in hen oviductal segments. VDR mRNA was first shown by Northern and slot blot analyses in the shell gland of laying hens, and it was observed that the level of *VDR* mRNA is closely related to eggshell calcification [36]. Further study demonstrated a strong VDR immunoreactivity in the mucosal epithelial and tubular gland cells, and a weak VDR immunoreactivity in stromal cells of the oviductal magnum, isthmus, and shell gland in immature, laying and molting hens [37]. VDR immunoreaction in the shell gland is stronger than in other oviductal sections and it is weaker in the shell gland of immature chickens than in laying and molting hens. In addition, two forms (~58 and 60 kDa) of VDR protein are present in the mucosal tissue of the shell gland of hens at different reproductive stages [37]; however, the importance of particular forms is not known. In the shell gland, about 2.0–2.5 g of calcium is deposited into the eggshell within a period of 11–15 h for the calcification of a single egg. Calcium is provided via the blood through trans-epithelial transport following absorption from the intestine or resorption from the bone [83,89]. There are lines of evidence indicating that vitamin D_3_ plays an essential role in the regulation of calcium metabolism and its role is mediated by VDR [83]. The main calcium-binding and intra-cellular transporting protein, calbindin D28K [90,91], expressed in the tubular gland cells of the shell gland is under the direct control of vitamin D_3_ and co-regulatory action of sex steroids in vitro [92]. Although the presence of a putative VDRE in the calbindin gene [93,94] further supports a possible role for vitamin D_3_, there is no evidence, in contrast to the intestine, of a direct in vivo effect of vitamin D_3_ on calbindin expression in the shell gland [83]. Another protein potentially stimulated by vitamin D_3_ is osteopontin, a multifunctional protein expressed by epithelial cells of the isthmus and shell gland, particularly during the period of eggshell calcification. Osteopontin is found in nonmineralized shell membrane fibers, the mammillary cores, and the outermost part of the palisade layer of the eggshell [95,96]. Additional studies are needed to fully understand local vitamin D_3_ roles, mechanisms of action, and regulation in the shell gland and other sections of the hen oviduct.

**Table 2:** Effects of vitamin D_3_ on the female reproductive system of farm animals and birds.

Tissue	Species	Cell Type	Treatment	Effect	References
*Ovary*	Goat	Gc	1, 10, 100 nM	↑ proliferation	[27,70]
	Goat	Gc	10 nM	↓ reactive oxygen species	[70]
	Goat	Gc	VDR silencing	↑ apoptosis	[70]
	Goat	Gc	10 nM	↑ P4 and E2	[27]
	Pig	SF, MF	10, 50 ng/mL	↑ P4	[29]
	Pig	Gc	100 nM	↓ P4	[74]
	Pig	Gc	100 ng/mL	→ P4	[73]
	Pig	Gc	100 ng/mL	↑ E2	[73]
	Pig	Gc	100 ng/mL	↑ E2	[74]
	Pig	SF	100 ng/mL	↑ E2	[29]
	Pig	MF	1–100 ng/mL	↑ E2	[29]
	Pig	SF, MF, LF	1–100 ng/mL	→ T	[29]
	Chicken	Gc	10, 100 nM	↑ proliferation	[30]
	Chicken	Gc	10, 100 nM	↓ *AMH* mRNA	[30]
	Chicken	Gc	100 nM	↑ *FSHR* mRNA	[30]
	Chicken	Gc	100 nM	↑ *Kit ligand* mRNA	[87]
	Chicken	-	vitamin D_3_-deficient diet	↓ E2 and P4	[86]
*Uterus*	Pig	M	10, 50 ng/mL	↑ E2	[33]
	Pig	E	2–200 µM	↑ implantation-related genes	[34]

Abbreviations: AMH = anti-Müellerian hormone; FSHR = follicle-stimulating hormone receptor; Gc = granulosa cells; E = endometrium; E2 = estradiol; LF = large follicles; M = myometrium; MF = medium follicles; P4 = progesterone; SF = small follicles; T = testosterone.

## 6. Vitamin D_3_ Action in the Female Reproductive System of Fish

The fish female reproductive tract in the majority of teleost species of aquaculture importance consists of paired ovaries, undergoing a seasonal reproductive cycle. This is comprised of two phases, gonadal recrudescence (long duration, several months) and oocyte final maturation and spawning (short duration, days). During the spawning season, the ovaries of mature female fish contain thousands or even millions of ready-to-release eggs, which is reflected in the gonadosomatic index (GSI), which is about 20–30% at this time, whereas in the recrudescence phase it is only 0.1–1% [97]. Most farmed fish are oviparous, so their embryos develop independently within an enclosed egg envelope (chorion) and rely on the compounds deposited within the oocytes during a few developmental stages from the primary ovarian follicle, then the cortical alveolus stage, vitellogenesis, and final oocyte maturation and ovulation [3]. 

The production of high-quality, fertilizable eggs that can further support successful embryonic, larval and later development of offspring is the main goal of modern aquaculture. The role of vitamin D_3_ in this important process and within the female fish reproductive system is not well known. However, the presence of VDR protein in the ovary of Atlantic salmon (*Salmo salar*) [44] and zebrafish (*Danio rerio*) [31] suggests the direct local actions of vitamin D_3_ on gonadal gametogenesis and steroidogenesis. The expression of VDR indicates that fish gonadal cells are a target for vitamin D_3_ action, and the proper level of 1α,25(OH)_2_D_3_ may be an important factor contributing to ovarian function. The role and mode of action of potentially locally produced vitamin D_3_ on fish gonadal maturation steroidogenesis and gametogenesis warrant further studies. 

## 7. Conclusions

**Figure 2:**
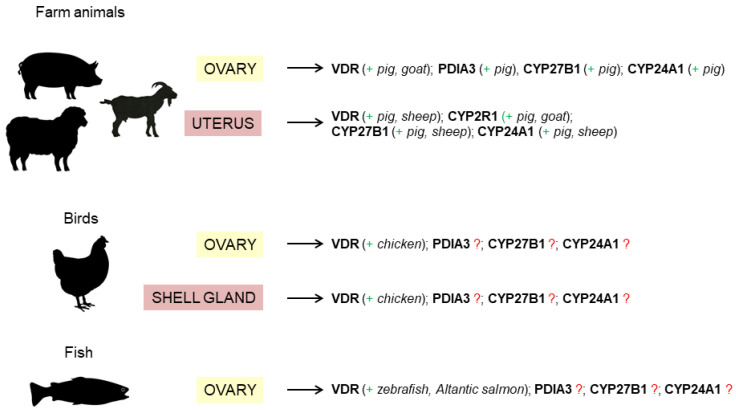
The known (+) and unknown (?) expression of vitamin D_3_ receptors and metabolic molecules in mammals, birds, and fish. Abbreviations: CYP2R1 = 25-hydroxylase; CYP27B1 = 1α-hydroxylase; CYP24A1 = 24-hydroxylase; PDIA3 = protein disulfide isomerase family A member 3; VDR = vitamin D_3_ receptor.

To summarize, the literature data indicate the plausible involvement of vitamin D_3_ in the regulation of reproductive processes in female farm animals via the interaction with its nuclear (VDR) and membranous (PDIA3) receptors detected in the ovary, uterus and placenta. Those reproductive organs are also a site of local vitamin D_3_ metabolism ensured by the enzymes CYP27B1 and CYP24A1. In birds, mainly the expression of VDR was noted in the ovary and oviduct, but there is a lack of information about vitamin D_3_ metabolism within the avian female reproductive tract. Taking into account that proper vitamin D_3_ status, especially in female farm animals and chickens, provides successful reproductive performance, it could be stated that its action within the reproductive system might be crucial for optimizing reproductive outcomes in female livestock. However, the least is known about fish (Figure 2). Knowing that there are differences in the cutaneous vitamin D_3_ metabolism between animal species that are often limited by indoor breeding [16], it is very important to provide effective dietary vitamin D_3_ supplementation that would cover the demand ensuring reproductive fitness. Finally, all the analyzed data suggest that additional studies are necessary to better understand the molecular mechanism of vitamin D_3_ action in the control of female fertility in livestock.

## Figures and Tables

**Table 1:** Vitamin D_3_-related molecule expression in the female reproductive system of farm animals, birds and fish.

Molecule	Tissue	Species	Compartment	mRNA	Protein (IHC/WB)	References
**VDR**	*Ovary*	Goat	Gc, Tc, O	+	+	[27]
		Pig	Gc, Tc	na	+	[28]
		Pig	Gc, Tc	+	+	[29]
		Chicken	Gc	+	+	[30]
		Fish	-	na	+	[31]
	*Uterus*	Buffalo cow	E	na	+	[32]
		Pig	E, M	+	+	[33]
		Pig	Gravid E	+	+	[34]
		Sheep	Gravid E, M	+	+	[35]
	*Shell gland*	Chicken	-	+	na	[36]
		Chicken	E, TG	na	+	[37]
	*Placenta*	Pig	-	+	+	[34]
		Sheep	-	+	+	[35]
**PDIA3**	*Ovary*	Pig	Gc, Tc	+	+	[29]
**CYP2R1**	*Uterus*	Pig	Gravid E	+	na	[34]
		Sheep	Gravid E, M	+	+	[35,38]
	*Placenta*	Pig	-	+	na	[34]
		Sheep	-	+	na	[35]
**CYP27B1**	*Ovary*	Pig	Gc, Tc	+	+	[29]
	*Uterus*	Pig	E, M	+	+	[39]
		Pig	Gravid E	+	na	[34]
		Sheep	Gravid E, M	+	+	[35,38]
	*Placenta*	Pig	-	+	na	[34]
		Sheep	-	+	na	[35]
**CYP24A1**	*Ovary*	Pig	Gc, Tc	+	+	[29]
	*Uterus*	Pig	E	+	+	[39]
		Pig	Gravid E	+	na	[34]
		Sheep	Gravid E	+	na	[35]
	*Placenta*	Pig	-	+	na	[34]
		Sheep	-	+	na	[35]

Abbreviations: CYP2R1 = 25-hydroxylase; CYP24A1 = 24-hydroxylase; CYP27B1 = 1α-hydroxylase; PDIA3 = protein disulfide isomerase family A member 3; VDR = vitamin D_3_ receptor; Gc = granulosa cells; Tc = theca cells; E = endometrium; TG = tubular glands; M = myometrium; O = oocyte; na = not assessed; IHC = immunohistochemistry; WB = Western blot.

## Data Availability

Not applicable.
